# Landscape of circulating metabolic fingerprinting for keloid

**DOI:** 10.3389/fimmu.2022.1005366

**Published:** 2022-09-29

**Authors:** Yu Hu, Xuyue Zhou, Lihao Chen, Rong Li, Shuang Jin, Lingxi Liu, Mei Ju, Chao Luan, Hongying Chen, Ziwei Wang, Dan Huang, Kun Chen, Jiaan Zhang

**Affiliations:** Institute of Dermatology, Jiangsu Key Laboratory of Molecular Biology for Skin Diseases and STIs, Chinese Academy of Medical Science & Peking Union Medical College, Nanjing, China

**Keywords:** keloid, metabolomics, risk score, mass spectrometry, biomarkers

## Abstract

**Background:**

Keloids are a fibroproliferative disease characterized by unsatisfactory therapeutic effects and a high recurrence rate.

**Objective:**

This study aimed to investigate keloid-related circulating metabolic signatures.

**Methods:**

Untargeted metabolomic analysis was performed to compare the metabolic features of 15 keloid patients with those of paired healthy volunteers in the discovery cohort. The circulating metabolic signatures were selected using the least absolute shrinkage. Furthermore, the selection operators were quantified using multiple reaction monitoring-based target metabolite detection methods in the training and test cohorts.

**Results:**

More than ten thousand metabolic features were consistently observed in all the plasma samples from the discovery cohort, and 30 significantly different metabolites were identified. Four differentially expressed metabolites including palmitoylcarnitine, sphingosine, phosphocholine, and phenylalanylisoleucine, were discovered to be related to keloid risk in the training and test cohorts. In addition, using linear and logistic regression models, the respective risk scores for keloids based on a 4-metabolite fingerprint classifier were established to distinguish keloids from healthy volunteers.

**Conclusions:**

In summary, our findings show that the characteristics of circulating metabolic fingerprinting manifest phenotypic variation in keloid onset.

## Background

Keloids are a benign fibroblast proliferative skin disorder characterized by an overabundance of fibroblasts beyond the boundaries of the original trauma (1–3). Several keloid treatments, including intradermal glucocorticoid injections, surgical excision, and radiation therapy, are available. However, therapeutic effects are usually unsatisfactory, with high recurrence rates (4). Notably, early identification of keloid-prone individuals is significantly important; this can minimize the occurrence of keloids by changing daily behaviors and modifying medical practices to avoid wounds as much as possible. However, it also helps timely intervention in the early stages of keloid formation to avoid serious adverse consequences.

Currently, the recognition of keloids relies heavily on the clinical presentation of keloids that typically extend beyond the original wound border. In addition, histopathological features of keloid collagen and α-smooth muscle actin contribute to diagnosis (1, 5). However, these diagnostic methods are not ideal owing to the need for invasive procedures, such as biopsy, because trauma could directly contribute to the formation of keloids. Consequently, there is an urgent need to explore new non-invasive methods and biomarkers for the early and accurate identification of keloids. For instance, liquid biopsies extracted by non-invasive methods from various biological fluids are performed to analyze the components, cells, or metabolites (6). With the advantages of being non-invasive, rapid, easy, and reproducible, it has become a useful tool for precision medicine, especially in the early diagnosis and individualized therapy of diseases.

Metabolites are intermediate products of metabolic reactions, including cellular biochemical and physiological processes, and are sensitive to internal and external environmental stimuli (7). Metabolomics, which is defined as the study of the collection of all small-molecule metabolites, could delineate characteristic metabolic “fingerprints” or “footprints” through targeted or non-targeted strategies, which contributes to the discovery of biomarkers with diagnostic, predictive, and therapeutic values (8). More importantly, several studies have suggested that keloid development is related to multiple metabolic pathways, such as glucose and lipid metabolism (9, 10). However, studies on body fluid biomarkers in keloid patients are lacking.

This study reports the landscape of circulating metabolic fingerprinting for keloids. Ultra-high-performance liquid chromatography coupled with Q Exactive mass spectrometry (UHPLC-QE-MS) platform was applied for untargeted metabolomic analysis in 15 keloid patients and healthy volunteers in a discovery cohort. Subsequently, the least absolute shrinkage and selection operator (LASSO) was used to screen four differentially expressed metabolites associated with the risk of keloids, including palmitoylcarnitine, sphingosine, phosphocholine, and phenylalanylisoleucine. These were validated through targeted metabolomic analysis in an expanded training and test cohort. Based on a 4-metabolite fingerprint classifier, we described the development and calculation of a risk score for recognizing keloids using a linear and logistic regression model. We propose that the characteristics of circulating metabolic fingerprinting could provide novel and convenient non-invasive testing targets and methods for the early identification of keloid-prone individuals.

## Methods

### Chemicals and reagents

High-performance liquid chromatography grade (HPLC)-grade acetonitrile (ACN) and methanol were supplied by Tedia Company Inc. (Fairfield, OH, USA). Formic acid (FA) was purchased from Sigma-Aldrich Co., Ltd. (St. Louis, MO, USA). Furthermore, phenylalanylisoleucine was purchased from Aladdin Company (Shanghai, China), phosphocholine from MedChemExpress (Shanghai, China), and palmitoylcarnitine and sphingosine from Sigma-Aldrich Co., Ltd. (St. Louis, MO, USA).

### Plasma sample preparation for metabolomics

Peripheral venous blood samples were drawn from fasting volunteers, and plasma was immediately separated and stored at -80°C. Sample preparation for untargeted metabolomics was performed according to the manufacturer’s instructions. First, one hundred microliters of each sample were slowly lysed at 4°C, after which 400 µL of pre-cooled methanol was added. Next, the samples were vortexed for 60 s, incubated at -80°C for 8 h, and the protein was precipitated by centrifugation at 14,000 rpm for 10 min at 4°C. Finally, the supernatant was used for HPLC analysis.

### Metabolite profiling analysis and metabolite identification

The samples were separated by ultra-high-performance liquid chromatography (UHPLC) and analyzed by mass spectrometry using a Thermo QE HF mass spectrometer (Thermo). Electrospray ionization (ESI)-positive and ESI-negative ion modes were used for detection. In addition, the MSdial program was used for peak data extraction, and the SIMCA program was used for principal component analysis (PCA) and orthogonal partial least-squares discrimination analysis (OPLS-DA). Subsequently, metabolite structure identification was performed using exact mass number matching, secondary spectrum matching, and public databases. Differential metabolites were categorized using the Human Metabolome Database (HMDB, http://www.hmdb.ca/).

### Metabolic network and pathway analysis

A correlation-based metabolic networking analysis was conducted after calculating Pearson’s correlation coefficient of the conversion of the signal intensity of significantly different metabolites by log transformation (Cytoscape 3.7, Institute of Systems Biology, Seattle). In addition, heat maps were obtained based on Spearman’s correlation and hierarchical clustering analysis. Metabolic pathways were analyzed using the KEGG database (http://www.genome.jp/kegg/pathway.html), and Fisher’s exact test was used to calculate the metabolite enrichment level of each pathway to identify the significantly affected metabolic and signaling pathways.

### Multiple reaction monitoring-based targeted metabolite method development

We performed an MRM-based targeted metabolite method according to a previously described protocol (11). Briefly, sample analysis was performed using the AB SCIEX ExionLC AD system coupled with the AB SCIEX QTRAP 5500 mass spectrometry system. Furthermore, LC separation was performed on an Agilent InfinityLab Poroshell 120 SB C18 column. The ESI MRM positive mode was selected to scan all peptides, and data were collected and analyzed using the AB SCIEX analyst software.

### Development of a risk score model for keloid

The plasma levels of candidate metabolites detected by the MRM-based targeted metabolite method were subjected to receiver operating characteristic (ROC) analysis to assess the sensitivity and specificity for distinguishing keloid patients from control patients. Linear and logistic regression patterns were established for the risk scores of patients with keloids.

### Statistical analysis

Results are presented as mean ± standard deviation (SD). Multidimensional statistical analysis was performed using SIMCA software, including unsupervised PCA and OPLS-DA. Volcano plots, heat maps, correlation string plots, and pathway bubble plots were drawn using R language software. The generally accepted level of significance was set at P < 0.05.

## Results

### Clinical features of subjects

**Figure 1:**
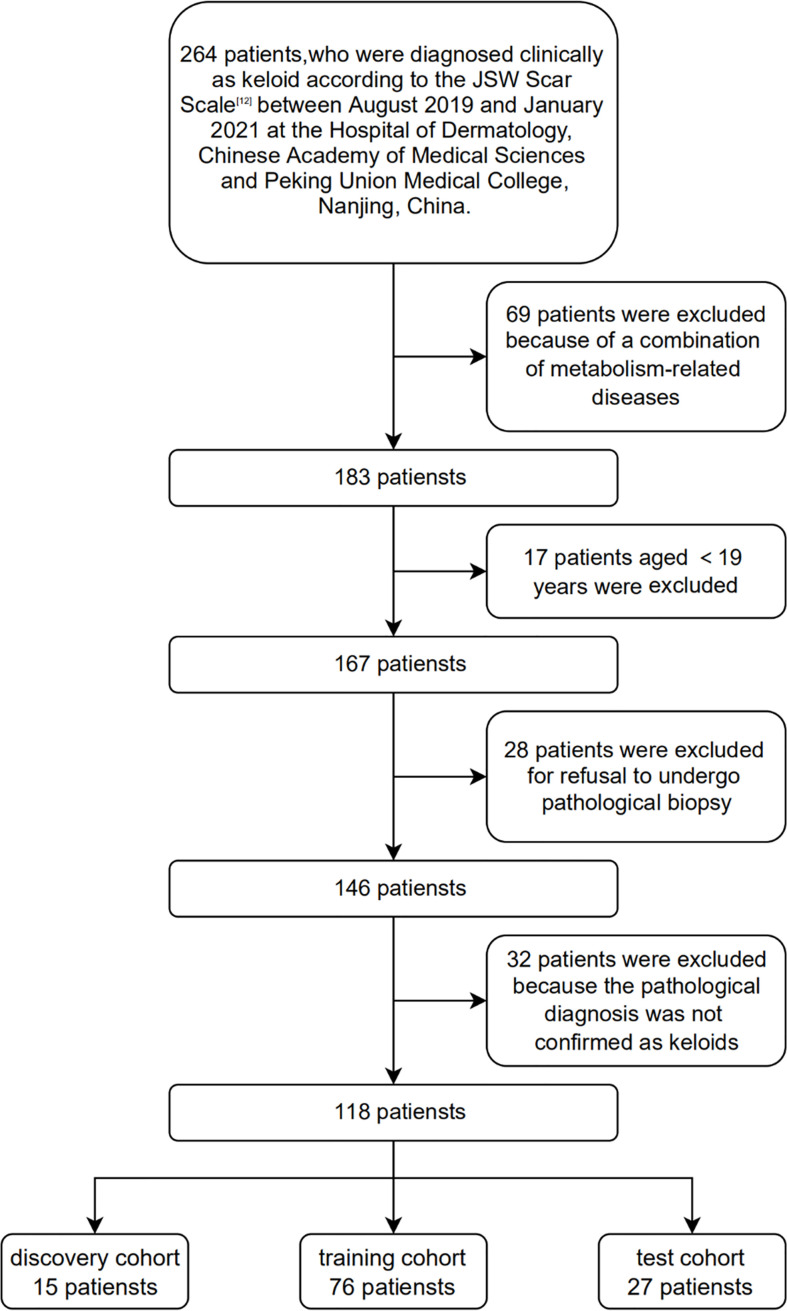
Flow chart of subject inclusion and exclusion criteria.

In this study, 118 patients with clinically and histopathologically confirmed keloids were recruited based on the diagnostic consensus of keloids (12) between August 2019 and January 2021 at the Hospital of Dermatology, Chinese Academy of Medical Sciences and Peking Union Medical College, Nanjing, China. One hundred and eighteen age- and sex-matched healthy volunteers were recruited as the controls. None of the control patients enrolled in this study developed keloids at their wound locations. In addition, none of the participants had a history of metabolic syndrome, obesity, dyslipidemia, hypertension, diabetes, or other metabolic diseases. 15, 76, and 27 plasma samples were selected as the discovery, training, and test cohorts, respectively. **Figure 1** shows the Flow chart of subject inclusion and exclusion criteria. **Table 1** presents the participants’ clinical features. The experiments were conducted in compliance with the Declaration of Helsinki, and the protocols were approved by the Institutional Ethical Review Board of Peking Union Medical College (No. 2021-KY-004). All participants were fully informed of the study details and provided written informed consent.

**Table 1:** Baseline clinical characteristics of subjects.

Characteristics of the subjects (*N* = 236)							
Characteristics		Discovery cohort (n = 30)		Training cohort (n = 152)		Test cohort (n = 54)	
		Keloid (n = 15)	Control (n = 15)	Keloid (n = 76)	Control (n = 76)	Keloid (n=27)	Control (n = 27)
**Age (years)**	**mean (SD)**	39.4 (12.5)	40.7 (13.4)	37.1 (13.9)	36.9 (13.3)	36.1 (11.1)	35.5 (12.2)
**Sex, n (%)**	**Female**	8 (53.3)	8 (53.3)	43 (56.6)	45 (59.2)	15 (55.6)	14 (51.2)
	**Male**	7 (46.7)	7 (46.7)	33 (43.4)	31(40.8)	12 (44.4)	13 (48.8)
**Duration (years)**	**mean (SD)**	6.9 (5.6)	NA	6.5 (4.9)	NA	6.6 (4.5)	NA
**Location**	**Face and neck**	1	NA	14	NA	3	NA
	**Trunk and shoulder**	13	NA	59	NA	22	NA
	**Extremities**	1	NA	3	NA	2	NA

NA, not applicable.

### The workflow of metabolomics

**Figure 2:**
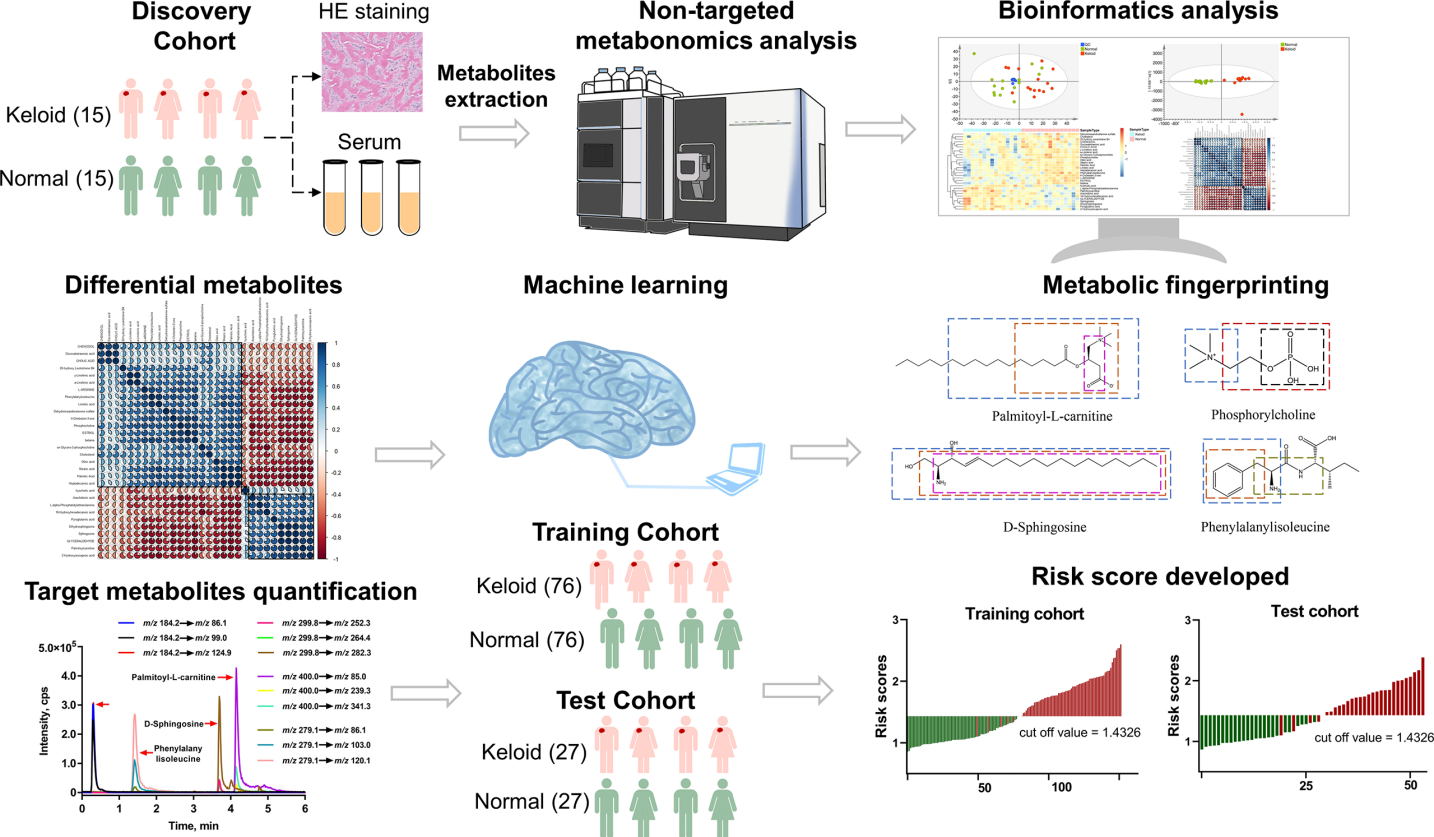
Workflow of the study design.

The research workflow of this study is illustrated in **Figure 2**. All patients in the discovery cohort were histopathologically confirmed to have keloids by HE staining. Plasma samples were collected from subjects in the discovery cohort and analyzed using the UHPLC-QE MS platform. Raw data were normalized using Pareto scaling for subsequent data analysis after extracting the background and aligning the metabolic peaks. Different metabolic features and metabolites were extracted by combining the criteria of fold change (FC) >1.2 and *P*<0.05, visualized with volcano plots and heat maps. In addition, thirty significantly different metabolites were screened using the threshold of variable importance for the projection (VIP) value >1 and P-value <0.05, for which correlation and pathway analyses were performed. Four metabolic predictors, including palmitoylcarnitine, sphingosine, phosphocholine, and phenylalanylisoleucine, were validated by MRM-based target metabolite quantification and analyzed in the training and test cohorts using LASSO analysis-based machine learning, which was conducted for feature selection. The risk scores for keloids based on a 4-metabolite fingerprint classifier were established.

### Metabolic profiling of keloid and control samples

**Figure 3:**
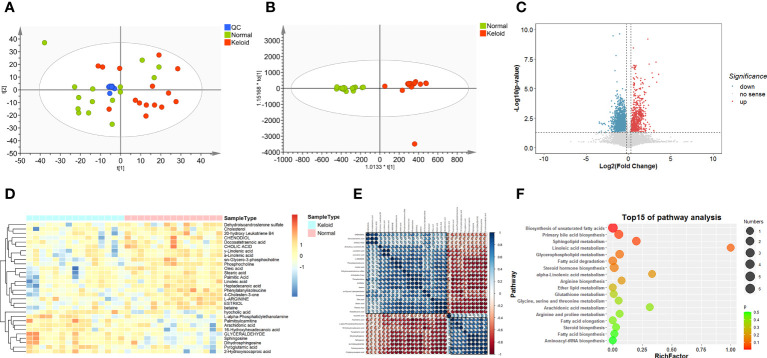
Metabolic profiling of keloid by mass spectrometry-based untargeted metabolomics analysis. **(A, B)** PCA and OPLS-DA score plots for control (green), keloid (red), and QC (blue) samples in ESI+ and ESI- models. **(C)** Volcano plot of differentially expressed metabolites between the keloid and control samples. (FC > 1.2, P < 0.05) Red dots indicate that metabolites are up-regulated in keloid patients, whereas blue dots indicate down-regulation. **(D)** Heatmap of the relative abundance of the metabolites differentially expressed between the keloid and control groups. **(E)** Pearson’s correlation coefficient analysis shows the metabolic network among 30 significantly different metabolites. **(F)** Metabolic pathway analysis of differential expressed metabolites.

We compared the metabolic signatures between the keloid and control groups in both ESI+ and ESI− modes of untargeted metabolomics. Overall, 15137 metabolic features were consistently found in all plasma samples from the discovery cohort, including 7062 features in ESI+ mode and 8075 in ESI– mode. Quality control (QC) samples were tightly clustered in PCA, validating the stability and reproducibility of instrumental analysis (**Figure 3A**). In ESI+ modes with R2Y = 0.962 and Q2 = 0.671 and in ESI- modes with R2Y = 0.941 and Q2 = 0.37, respectively; the OPLS-DA score plot showed a clear demarcation between the keloid and control groups, indicating significant changes in plasma metabolites in keloid patients (**Figure 3B**).

### Identification of differential metabolites

We calculated the P-values and fold changes for all metabolic features in keloids and control samples, which were visualized using volcano plots (**Figure 3C**). Differentially regulated metabolic features were investigated with the criteria of FC > 1.2 and P-value < 0.05. Subsequently, 30 significantly different metabolites were further filtered by the threshold of VIP value > 1 and P-value < 0.05, containing 11 and 19 metabolites from the ESI+ and ESI− models, respectively. They were classified into five major categories and 14 subcategories according to the chemical taxonomy in the Human Metabolome Database (HMDB) (13) (**Table 2**). In addition, hierarchical clustering analysis revealed a different metabolic pattern between keloids and control samples (**Figure 3D**). Pearson’s correlation coefficient analysis was used to analyze the metabolite-metabolite correlation among the identified metabolites, showing a metabolic network among these 30 significantly different metabolites (**Figure 3E**). Finally, KEGG pathway enrichment analysis was performed for 30 dysregulated metabolites involving 15 metabolic pathways. Based on the rich factor and P-value, biosynthesis of unsaturated fatty acids, primary bile acid biosynthesis, sphingolipid metabolism, linoleic acid metabolism and glycerophospholipid metabolism were the most significantly enriched metabolic pathways (**Figure 3F**).

**Table 2:** Significantly altered metabolites in keloid vs. normal controls in the analysis of ESI+ mode and ESI- mode.

Fatty Acyls	Fatty acids and conjugates	16-Hydroxyhexadecanoic acid	1.46	0.04	1.25	ESI-
		2-Hydroxyisocaproic acid	1.67	0	1.31	ESI-
		Arachidonic acid	1.57	0.01	1.07	ESI-
		Docosatetraenoic acid	0.7	0.05	1.02	ESI-
		Heptadecanoic acid	0.63	0.01	1.13	ESI-
		Oleic acid	0.7	0.01	1.01	ESI-
		Palmitic Acid	0.64	0.01	1.2	ESI-
		Stearic acid	0.62	0	1.31	ESI-
	Lineolic acids and derivatives	α-Linolenic acid	0.56	0.01	1.71	ESI-
		Linoleic acid	0.53	0.02	1.58	ESI-
		γ-Linolenic acid	0.61	0.03	1.39	ESI-
	Eicosanoids	20-hydroxy Leukotriene B4	0.61	0.01	1.33	ESI-
	Fatty acid esters	Palmitoylcarnitine	1.61	0	1.49	ESI+
Steroids and steroid derivatives	Bile acids, alcohols and derivatives	Chenodiol	0.49	0.05	1.61	ESI-
		Cholic acid	0.63	0.04	1.06	ESI-
		hyocholic acid	1.23	0.01	1	ESI+
	Cholestane steroids	4-Cholesten-3-one	0.74	0.01	1.09	ESI+
		Cholesterol	0.64	0.04	1	ESI+
	Sulfated steroids	Dehydroisoandrosterone sulfate	0.52	0.01	1.52	ESI-
	Estrane steroids	Estriol	0.43	0	1.62	ESI-
Organooxygen compounds	Amines	Dihydrosphingosine	1.5	0.03	1.16	ESI+
		Sphingosine	2.51	0	1.91	ESI+
	Carbohydrates and carbohydrate conjugates	Glyceraldehyde	2.46	0	1.52	ESI-
	Quaternary ammonium salts	Phosphocholine	0.71	0	1.26	ESI+
Carboxylic acids and derivatives	Amino acids, peptides, and analogues	Pyroglutamic acid	1.57	0.02	1.2	ESI-
		betaine	0.75	0	1.27	ESI+
		L-arginine	0.72	0.02	1.1	ESI+
		Phenylalanylisoleucine	0.71	0	1.24	ESI+
Glycerophospholipids	Glycerophosphoethanolamines	L-alpha-Phosphatidylethanolamine (Soy)	2.05	0.04	2.21	ESI-
	Glycerophosphocholines	sn-Glycero-3-phosphocholine	0.45	0.01	1.58	ESI+

### Metabolic fingerprinting and target metabolites quantification

**Figure 4:**
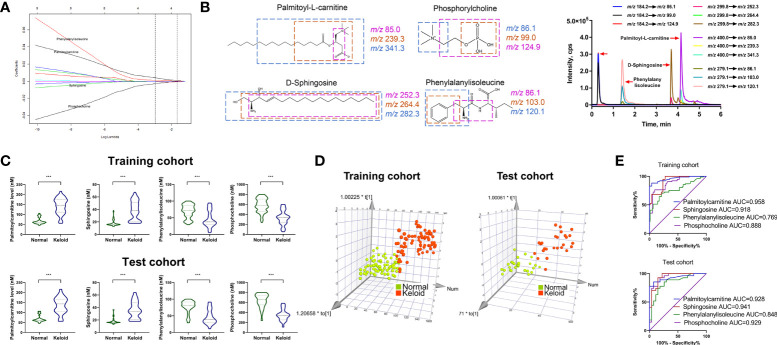
Development of MRM-based target metabolite quantification method and detection of the abundance of four target metabolites. **(A)** LASSO coefficient profiles of the nine features. **(B)** Chemical structure, fragmentation, and representative MRM chromatograms of palmitoylcarnitine, sphingosine, phosphocholine, and phenylalanylisoleucine. **(C, D)** Respective expression of palmitoylcarnitine, sphingosine, phosphocholine, and phenylalanylisoleucine and the 3D PCA score plot based on the 4-metabolite fingerprint classifier in the training and test cohorts. **(E)** ROC analysis of palmitoylcarnitine, sphingosine, phosphocholine, and phenylalanylisoleucine in predicting keloid in the training and test cohorts. *p < 0.05 and ***p < 0.001.

The LASSO regression model was utilized to select biomarkers to identify keloids *via* penalized maximum likelihood, providing the most regularized model in applying four markers. Four metabolites were selected as potential predictors from the significantly different metabolites in the ESI+ model based on the minimum criteria with one standard error (the 1-SE criteria) of the values for the regularization parameter lambda (**Figure 4A**). Subsequently, we validated four metabolite signatures, palmitoylcarnitine, sphingosine, phosphocholine, and phenylalanylisoleucine, in the training and test cohorts using MRM-based targeted metabolite analysis. The levels of palmitoylcarnitine, sphingosine, phosphocholine, and phenylalanylisoleucine were monitored in three MRM transitions: *m/z* 400.0→85.0, 400.0→239.3, and 400.0→341.3; 299.8→252.3, 299.8→264.4, and 299.8→282.3; 184.2→86.1, 184.2→99.0, and 184.2→124.9; and 279.1→86.1, 279.1→103.0, and 279.1→120.1, respectively (**Figure 4B**). As shown in **Figure 4C**, palmitoylcarnitine and sphingosine plasma levels were higher in the keloid group than in the normal group, whereas phosphocholine and phenylalanylisoleucine levels decreased (*P* < 0.001).

Furthermore, multivariate analysis was performed to visualize the cross-group comparisons. The 3D PCA score plot with the keloid and control groups showed that the keloid and control groups clustered in different directions based on a 4-metabolite fingerprint classifier (**Figure 4D**). Finally, we performed a ROC curve analysis to evaluate the power of these four differentially expressed metabolites in predicting keloids. The result (**Figure 4E**) displays the ROC curve with an area under the curve (AUC) of 0.958, 0.918, 0.888, and 0.769 for palmitoylcarnitine, sphingosine, phosphocholine, and phenylalanylisoleucine, respectively, in the training cohort. In contrast, they were 0.928, 0.941, 0.848, and 0.929 for palmitoylcarnitine, sphingosine, phosphocholine, and phenylalanylisoleucine, respectively, in the test cohort.

### Identification of keloid based on 4-metabolite fingerprint classifier

**Figure 5:**
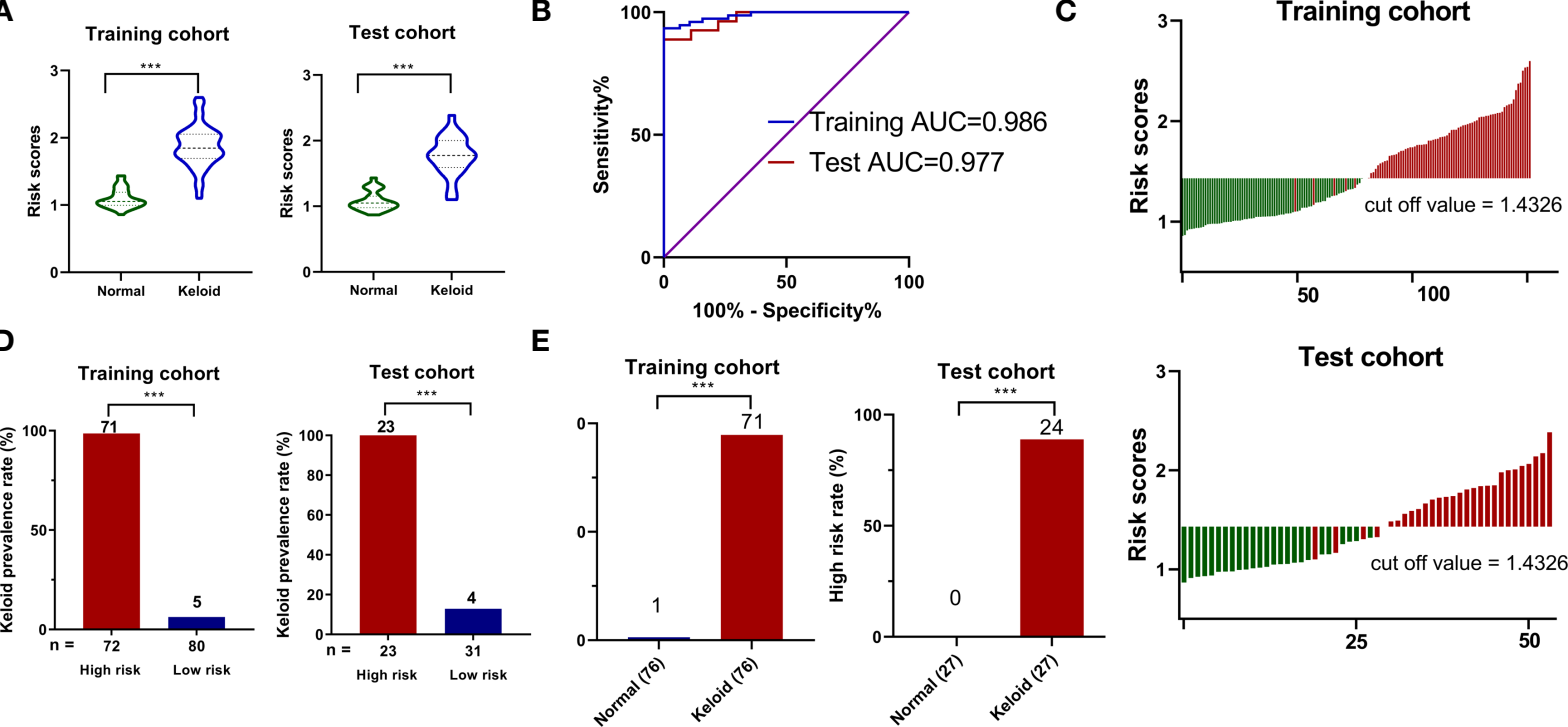
The construction of risk score model for identifying keloid. **(A)** Respective values of keloid risk scores between the keloid and control groups in the training and test cohorts. **(B)** ROC analysis of sepsis risk scores in predicting keloid. **(C)** An optimal threshold point value was defined as 1.4326, and all participants were classified into low-risk (<cutoff) and high-risk (>cutoff) score groups. **(D)** Analysis of keloid prevalence rate between the high-risk and low-risk groups in the training and test cohorts. **(E)** Analysis of high-risk rate between the keloid and control groups in the training and test cohorts. ***p < 0.001.

We calculated the characteristic risk score to elucidate further the combined index of palmitoylcarnitine, sphingosine, phosphocholine, and phenylalanylisoleucine to diagnose keloids. In addition, we built a logistic regression model comprising these four biomarkers in the training and test cohorts. The equation for the probability of keloid occurrence can be defined as follows: Ln (keloid risk score) = 0.6508 × Ln (palmitoylcarnitine) + 0.03997× Ln (phenylalanylisoleucine) -0.3486 × Ln (phosphocholine) + 0.1228 × Ln (sphingosine) – 0.9183. The risk scores in the keloid group were considerably higher than those in the normal group in both cohorts (**Figure 5A**). Furthermore, ROC analysis of the model built with 4-metabolite fingerprint classifier yielded a sensitivity and specificity of 93.4% and 100.0% (AUC = 0.986, P < 0.001) in the training cohort and 88.9% and 100.0% (AUC = 0.977, P < 0.001) in the test cohort, respectively (**Figure 5B**), suggesting the predictive ability of our novel score. The optimal threshold point value was defined as 1.4326, using the Youden index in the training cohort. Also, all participants were classified into low-(<cutoff) and high-risk (>cutoff) score groups using the 4-metabolite fingerprint classifier (**Figure 5C**). The prevalence of keloid was significantly higher in the high-risk groups (98.6% and 100%) than in the low-risk groups (6.25% and 12.9%) in the training and test cohorts, respectively (**Figure 5D**). In addition, the rate of high risk was higher in the keloid group than in the control group in both the training and test cohorts. The high-risk rates of the keloid group (93.4% and 88.8%) were significantly higher than those of the normal group (1.3% and 0%) in the training and test cohorts, respectively (**Figure 5E**).

## Discussion

To date, the accurate identification of keloids relies on the clinical presentation and histopathology of the biopsied tissue of the formed or mature keloids. Current keloid therapies, such as intralesional corticosteroid injections, surgical excision, and postoperative radiation therapy, have limited efficacy and a high risk of recurrence. However, given the difficulty in treating keloid scars, more emphasis should be placed on the early identification and prevention of keloid management. Therefore, developing non-invasive, specific, and sensitive tools that can be used for early keloid identification is extremely important. Metabolomics has emerged as a promising technology for non-invasive evaluation using biofluids such as blood and urine. In addition, it is cheaper than other omics methods such as proteomics and transcriptomics, making it easier to translate into clinical practice (14).

This study investigated and identified the landscape of metabolic fingerprinting for the identification and risk scoring of keloids. We performed an untargeted metabolomic analysis based on the UHPLC-QE-MS platform to characterize the metabolic profiles of patients with keloids. Thirty statistically different metabolites were identified, most of which were fatty acyl groups, steroids and steroid derivatives, organooxygen compounds, carboxylic acids and derivatives, and glycerophospholipids, which is consistent with previous studies. Zhang *et al.* performed a gene microarray analysis on fibroblasts from normal, hypertrophic scars, and keloid tissues. The results suggested that differentially expressed genes were strongly associated with sterol, fatty acid, and steroid metabolic processes (15). Compared to the normal human epidermis, the number of triglycerides, cholesteryl ester, and wax ester decreased by 60%, 33%, and 80% in the epidermis of keloid lesions, respectively (16). Consistently, our study also found that cholesterol levels decreased in the plasma of keloid patients detected in ESI+ mode. In addition, lipid extraction, fractionation, and fatty acid identification of skin lesions from African keloid patients revealed a deficiency of essential fatty acids, such as linoleic acid, γ-linolenic acid, dihomo-γ-linolenic acid, α-linolenic acid, and eicosapentaenoic acid. However, it showed elevated levels of arachidonic acid in keloids (17). In the untargeted metabolomic analysis, α-linolenic acid, linoleic acid, and γ-linolenic acid were significantly down-regulated metabolites in the keloid plasma. In contrast, arachidonic acid was significantly up-regulated. α-Linolenic acid dose-dependently inhibits the growth of fibroblasts in keloid patients, suggesting a protective effect on keloid formation (18). However, the decline in α-linolenic acid in the plasma and skin lesions of keloid patients may contribute to keloid susceptibility. Therefore, the release of [3H]arachidonic acid from keloid-derived fibroblasts was greater than that from normal skin-derived fibroblasts (19). Arachidonic acid is pro-inflammatory, and its downstream products, such as leukotrienes, prostaglandins, prostacyclins, and thromboxane, are considered to be pro-inflammatory modulators (9).

We identified and confirmed for the first time using the LASSO logistic regression model that up-regulated levels of palmitoylcarnitine and sphingosine, and down-regulated levels of phosphocholine and phenylalanylisoleucine are strongly correlated with the early identification of keloids. High levels of plasma palmitoylcarnitine have been observed in various cancers and chronic diseases such as obesity and type 2 diabetes (20–22). Palmitoylcarnitine has been found to induce Ca^2+^ influx and promote the secretion of the pro-inflammatory factor IL-6 (23). GO analysis of lncRNA expression profiles in keloid tissues revealed a vital role in calcium pathway signaling, and calcium channel blockers have been reported to treat keloids (24, 25). IL-6 has been shown to promote proliferation and collagen synthesis in keloid-derived fibroblasts, thus participating in keloid pathogenesis (26). Therefore, we hypothesized that palmitoylcarnitine plays a potential role in promoting keloid formation. Sphingosine is a classic product of sphingolipid metabolism. Together with ceramide, sphingosine-1-phosphate is fundamental in regulating cell proliferation, survival, and death. Although ceramide and sphingosine generally suppress proliferation and promote apoptosis, sphingosine-1-phosphate is associated with growth stimulation and apoptosis inhibition; hence, there is a “sphingolipid rheostat” effect between ceramide, sphingosine, and sphingosine-1-phosphate, indicating that these metabolites are interconvertible. Notably, the fate of cells is determined by their relative levels rather than their absolute amounts of these metabolites (27). Sphingosine and sphingosine-1-phosphate have been reported to promote cell proliferation at low concentrations through calcium metabolism and activation of the MAPK pathway in Swiss 3T3 cells; however, they are cytotoxic at high concentrations (28). Fingolimod (FTY720), an analog of sphingosine-1-phosphate, has been used to treat keloid scars (29). Therefore, we hypothesized that elevated sphingosine levels in keloid plasma might be involved in keloid development by affecting the relative concentration of sphingosine-1-phosphate. Phosphocholine is the polar headgroup of the dominant membrane phospholipid phosphatidylcholine. In addition, bioactive phosphocholine-containing lipids can be recognized by the innate immune system and play a crucial role in endothelial dysfunction, apoptosis, and endoplasmic reticulum stress (30). Phenylalanylisoleucine is a dipeptide composed of phenylalanine and isoleucine and is an incomplete breakdown product of protein digestion or catabolism. Dysregulation of amino acid metabolism is associated with protein synthesis, glucose and lipid homeostasis, mTOR signalling pathways, and immune responses (31). Although the specific mechanisms of these four metabolite predictors is yet to be elucidated in the pathogenesis of keloids, our findings highlight the possible clinical application of plasma metabolic markers in predicting keloid formation.

There are certain limitations to this study. Therefore, future prospective multicenter studies with large size of samples from different periods of keloids are required to further validate the diagnostic model’s specificity and sensitivity. In addition, complete coverage of the keloid metabolome by detecting and identifying all metabolites remains challenging and requires the application of different detection platforms and the development of metabolomic databases.

## Conclusions

In summary, we successfully determined the plasma metabolic profile of keloid patients and developed a risk score model for keloid identification based on the 4-metabolite fingerprint classifier in plasma. We hope that this study will aid the development of non-invasive and early identification or prediction tools for keloids, thus opening up new possibilities for the early personalized treatment of keloid patients.

## Data availability statement

The raw data supporting the conclusions of this article will be made available by the authors, without undue reservation.

## Ethics statement

The studies involving human participants were reviewed and approved by Institutional Ethical Review Board of Peking Union Medical College. The patients/participants provided their written informed consent to participate in this study.

## Author contributions

DH, KC and JZ conceived and designed the project. YH, XZ, RL, SJ and LL performed the experiments. MJ, CL, HC, LC and ZW analyzed experimental results. YH and XZ wrote the manuscript with approval from all authors. DH, KC and JZ revised the manuscript. All authors contributed to the article and approved the submitted version.

## Funding

This study is supported by grants from National Natural Science Foundation of China (No. 82273552, 82203947 and 82073445), Natural Science Foundation of Jiangsu Province (No. BK20210049), CAMS Innovation Fund for Medical Sciences (CIFMS-2021-I2M-1-001) and Medical Scientific Research Project of Jiangsu Provincial Health Commission (M2022113).

## Conflict of interest

The authors declare that the research was conducted in the absence of any commercial or financial relationships that could be construed as a potential conflict of interest.

## Publisher’s note

All claims expressed in this article are solely those of the authors and do not necessarily represent those of their affiliated organizations, or those of the publisher, the editors and the reviewers. Any product that may be evaluated in this article, or claim that may be made by its manufacturer, is not guaranteed or endorsed by the publisher.
